# Follow-up strategies after trimodal treatment for muscle-invasive bladder cancer: a systematic review

**DOI:** 10.1007/s00345-024-05196-7

**Published:** 2024-09-19

**Authors:** Ernest Kaufmann, Stefanie Aeppli, Winfried Arnold, Panagiotis Balermpas, Jörg Beyer, Uwe Bieri, Richard Cathomas, Berardino de Bari, Marco Dressler, Daniel S. Engeler, Andreas Erdmann, Andrea Gallina, Silvia Gomez, Matthias Guckenberger, Thomas R. W. Herrmann, Thomas Hermanns, Lucca Ilaria, Hubert John, Thomas M. Kessler, Jan Klein, Mohamed Laouiti, David Lauffer, Agostino Mattei, Michael Müntener, Daniel Nguyen, Philipp Niederberger, Alexandros Papachristofilou, Lukas Prause, Karsten Reinhardt, Emanuela Salati, Philippe Sèbe, Mohamed Shelan, Räto Strebel, Arnoud J. Templeton, Ursula Vogl, Marian S. Wettstein, Deborah Zihler, Thomas Zilli, Daniel Zwahlen, Beat Roth, Christian Fankhauser

**Affiliations:** 1 Department of Urology, University of Lucerne, Luzerner Kantonsspital, Spitalstrasse, 6000, 16, Lucerne, Switzerland; 2 Department of Oncology, Kantonsspital St. Gallen, St. Gallen, Switzerland; 3 Department of Radiation-Oncology, Luzerner Kantonsspital, Lucerne, Switzerland; 4 Department of Radiation-Oncology, Universitiy Hospital Zurich, Zurich, Switzerland; 5 Department of Oncology, Inselspital Bern, Berne, Switzerland; 6 Department of Urology, Kantonsspital Baden, Baden, Switzerland; 7 Department of Urology, University Hospital Zurich, Zurich, Switzerland; 8 Department of Oncology, Kantonsspital Chur, Chur, Switzerland; 9 Department of Radiation-Oncology, Réseau Hospitalier Neuchâtelois, Neuchâtel, Switzerland; 10 Zentrum Für Onkologie Luzern, Lucerne, Switzerland; 11 Department of Urology, Kantonsspital St. Gallen, St. Gallen, Switzerland; 12 Department of Oncology, Kantonsspital Baden, Baden, Switzerland; 13 Department of Urology, EOC Ente Ospedaliero Cantonale, Lugano, Switzerland; 14 Department of Radiation-Oncology, Kantonsspital Aarau, Aarau, Switzerland; 15 Department of Urology, Spital Thurgau AG, Kantonsspital Frauenfeld, Frauenfeld, Switzerland; 16 Division of Urology, Department of Surgical Sciences, Stellenbosch University, Western Cape, South Africa; 17 Hannover Medical School, Hannover, Germany; 18 Zentrum für Urologie Zürich, Zurich, Switzerland; 19 Department of Urology, Centre Hospitalier Universitaire Vaudois (CHUV), Lausanne, Switzerland; 20 Department of Urology, Kantonsspital Winterthur, Winterthur, Switzerland; 21 Department of Neuro-Urology, Balgrist University Hospital, University of Zurich, Zurich, Switzerland; 22 Department of Urology, Kantonsspital Münsterlingen, Müsterlingen, Switzerland; 23 Department of Urology, Medical School, Ulm, Germany; 24 Department of Radiation-Oncology, Hôpital Riviera Chablais, Rennaz, Switzerland; 25 Department of Radiation-Oncology, University Hospital Geneva, Geneva, Switzerland; 26 Department of Urology, Stadtspital Triemli, Zurich, Switzerland; 27 Department of Urology, Réseau Hospitalier Neuchâtelois, Neuchâtel, Switzerland; 28 Department of Oncology, Luzerner Kantonsspital, Lucerne, Switzerland; 29 Department of Radiation-Oncology, University Hospital Basel, Basel, Switzerland; 30 Department of Urology, Kantonsspital Aarau, Aarau, Switzerland; 31 Department of Urology, St. Clara Hospital Basel, Basel, Switzerland; 32 Department of Oncology, Hôpital Riviera Chablais, Rennaz, Switzerland; 33 Department of Urology, University Hospital Geneva, Geneva, Switzerland; 34 Department of Radiation-Oncology, Inselspital Bern, Berne, Switzerland; 35 Department of Urology, Kantonsspital Chur, Chur, Switzerland; 36 Department of Oncology, St. Claraspital Basel and Faculty of Medicine, University Basel, Basel, Switzerland; 37 Department of Oncology, EOC Ente Ospedaliero Cantonale, Bellinzona, Switzerland; 38 Department of Uro-Oncology, University of Toronto, Toronto, Canada; 39 Department of Oncology, Kantonsspital Aarau, Aarau, Switzerland; 40 Department of Radiation-Oncology, EOC Ente Ospedaliero Cantonale, Bellinzona, Switzerland; 41 Department of Radiation-Oncology, Kantonsspital Winterthur, Winterthur, Switzerland; 42 Department of Urology, Inselspital Bern, Berne, Switzerland

**Keywords:** Trimodal treatment, Trimodality, Bladder preservation, Functional outcomes, Oncological outcomes, Follow-up

## Abstract

**Purpose:**

Optimal follow-up strategies following trimodal treatment for muscle invasive bladder cancer play a crucial role in detecting and managing relapse and side-effects. This article provides a comprehensive summary of the patterns and risk factors of relapse, functional outcomes, and follow-up protocols.

**Methods:**

A systematic literature search on PubMed and review of current guidelines and institutional follow-up protocols after trimodal therapy were conducted.

**Results:**

Out of 200 identified publications, 43 studies (28 retrospective, 15 prospective) were selected, encompassing 7447 patients (study sizes from 24 to 728 patients). Recurrence rates in the urinary bladder varied between 14–52%; 3–16% were muscle-invasive while 11–36% were non-muscle invasive. Nodal recurrence occurred at 13–16% and distant metastases at 15–35%. After 5 and 10 years of follow-up, around 60–85% and 45–75% of patients could preserve their bladder, respectively. Various prognostic risk factors associated with relapse and inferior survival were proposed, including higher disease stage (> c/pT2), presence of extensive/multifocal carcinoma in situ (CIS), hydronephrosis, multifocality, histological subtypes, incomplete transurethral resection of bladder tumor (TURBT) and incomplete response to radio-chemotherapy. The analyzed follow-up guidelines varied slightly in terms of the number, timing, and types of investigations, but overall, the recommendations were similar.

**Conclusion:**

Randomized prospective studies should focus on evaluating the impact of specific follow-up protocols on oncological and functional outcomes following trimodal treatment for muscle-invasive bladder cancer. It is crucial to evaluate personalized adaption of follow-up protocols based on established risk factors, as there is potential for improved patient outcomes and resource allocation.

**Supplementary Information:**

The online version contains supplementary material available at 10.1007/s00345-024-05196-7.

## Introduction

Bladder cancer poses a significant public health challenge, with approximately 500,000 new cases and 200,000 deaths reported annually worldwide [1]. Radical cystectomy represents the most commonly performed therapy for muscle-invasive bladder cancer but may be associated with relevant morbidity and functional impairment leading to a profound impact on patient’s quality of life [2, 3]. Trimodal treatment, consisting of transurethral resection of bladder tumor (TURBT) followed by chemoradiotherapy, has emerged as an alternative approach in selected patients [4]. Numerous studies have suggested comparable long-term survival outcomes between trimodal treatment and radical cystectomy while mitigating perioperative morbidity and preserving patient’s quality of life by bladder preservation [5–10]. With improved treatment protocols, up to 85% of all patients with muscle-invasive bladder cancer can preserve their bladder at 5 years after trimodal treatment [8, 10].

Following trimodal treatment, rigorous post-treatment surveillance is imperative to detect recurrence promptly and allow bladder preservation by repeated TURBT or salvage cystectomy. Monitoring for functional impairments and adverse effects attributed to chemoradiation is also important during and subsequent to the therapy. Presently, clinical evidence guiding the optimal follow-up after trimodal treatment is sparse, with current guidelines largely predicated on consensus from experts. Additionally, while radical cystectomy is associated with marginally elevated costs related to the surgical procedure, the costs of trimodal treatment increase during follow-up, mainly driven by the costs of cystoscopic monitoring [11–14]. Therefore, there is a need to develop tailored surveillance strategies that align with specific treatment modalities and risk profiles, aiming to optimize healthcare expenditure by mitigating redundant diagnostic procedures during follow-up. In this systematic review, we provide a comprehensive summary of the proportion, timing, and anatomical sites of relapse following trimodal treatment, along with the functional complications/toxicities associated with the treatment. Furthermore, we discuss published follow-up protocols and explore current and future developments in the follow-up care of patients who have undergone trimodal treatment.

### Evidence acquisition

We conducted a systematic literature search on PubMed on February 22, 2024, in accordance with the Preferred Reporting Items for Systematic Review and Meta-Analysis (PRISMA) statement to identify published follow-up protocols for patients who have undergone trimodal treatment for bladder cancer (see Supplementary File 1). This systematic review was registered with PROSPERO, the international prospective register for systematic reviews (registration number: CRD42024525165).

Our search included (1) current guidelines from the American Urology, Oncology and Radiation Oncology Associations (AUA/ASCO/ASTRO/SUO), Bladder Cancer Canada, the European Association of Urology (EAU), the European Society for Medical Oncology (ESMO), the German S3-guideline, the National Comprehensive Cancer Network (NCCN) and (2) follow-up protocols of published networks or institutions. We excluded non-English literature (with exception of the German S3-Guidelines) and publications before 1990. We manually screened the reference lists of identified publications to find additional relevant studies, and we used Endnote’s ‘close match’ function to filter out duplicate articles. One investigator (EK) screened all titles, abstracts, and full texts for inclusion in the study. Data from the same study that appeared in multiple publications were only included once. Finally, we only included published manuscripts containing a minimum of 20 patients in the analysis. To reduce risk of bias and resolve any discrepancies, a second investigator (CDF) was involved in the screening process.

### Evidence synthesis

We identified 200 publications that met our initial search criteria, and we conducted a title and abstract screening of 92 full texts (Supplementary Fig. 1). After screening, we included 43 studies in our analysis, of which 28 were retrospective and 15 were prospective (see Supplementary Table 1). These 43 studies included a range of 24–728 patients, providing data for a total of 7447 patients.

### Optimal diagnostic testing for oncological follow-up

The primary objective of oncological follow-up after trimodal treatment is to identify any signs of local or systemic recurrence and to screen for secondary malignancies that are frequently associated with bladder cancer, such as urothelial carcinomas of the urethra or upper urinary tract, as well as lung cancer, particularly in individuals who are current or former smokers. Given the variability in accuracy among different imaging modalities in detecting these recurrences, current guidelines advocate for the use of multiple modalities in follow-up imaging. Cystoscopy and cytology are recommended for detecting local recurrences, while cross-sectional imaging techniques such as computerized tomography (CT), magnetic resonance imaging (MRI), or 18F-fluorodeoxyglucose positron emission tomography (FDG-PET-CT) are recommended to identify both local recurrence and distant metastases [15].

### Cross-sectional imaging

No comparative studies comparing different cross-sectional imaging modalities or specific time-points for follow-up after trimodal treatment for muscle-invasive bladder cancer have been identified. As a result, most guidelines for follow-up protocols are derived from cystectomy follow-up protocols [16]. The current recommendations of recognized guidelines are summarized in Table 1. The American Urological Association (AUA) suggests CT or MRI of the abdomen and pelvis, along with chest imaging, every 6 months during the first 2 years, and subsequently on an annual basis [17]. According to the European Association of Urology (EAU) and the European Society for Medical Oncology (ESMO), CT of the chest and abdomen should be conducted every 3–4 months during the first 2 years, followed by imaging every 6–12 months until the 5th year [18, 19]. As for the German S3-Guidelines, they recommend CT of the chest and abdomen every 6 months for 2–3 years, followed by annual imaging until the 5th year [20]. The National Comprehensive Cancer Network (NCCN) recommends CT urography (CTU) or MR urography (MRU) of the abdomen and pelvis, including thoracic CT, every 3–6 months in the first 2 years, and then annually until the 5th year [15]. It should be mentioned that only the NCCN recommends FDG-PET-CT in their guidelines.

**Table 1:** Comparison of different oncological follow-up schedules including investigations and their intervals in between (in months) after trimodal therapy for bladder cancer

MIBC	Months after TMT	3	6	9	12	15	18	21	24	27	30	33	36	42	48	54	60	61–120
AUA	visit	(x)	x	(x)	x	(x)	x	(x)	x				x		x		x	
	CT Chest		x		x		x		x									
	CT or MRI Abd/Pelv		x		x		x		x									
	Cystoscopy	x	x	x	x	(x)	x	(x)	x		(x)		x	(x)	x	(x)	x	
	Cytology	?																
BLADDER CANCER CANADA	visit	x	x	x	x	x	x	x	x		x		x	x	x		x	Annually
	chest x-ray or CT Thorax		x’		x		x’		x		x´´		x	x´´	x	x´´	x	
	CT Abdomen		x’		x		x’		x				x		x’		x	
	Cystoscopy	x	x	x	x	x	x	x	x		x		x	x	x		x	Annually
	Cytology				(x)				(x)				(x)		(x)		(x)	
EAU	visit	x	x	x	x	x	x	x	x	x	x	x	x	x	x	x	x	
	CT Chest		x		x		x		x				x		x		x	
	CT Abd/Pelv		x		x		x		x				x		x		x	
	Cystoscopy	x	x	x	x	x	x	x	x	x	x	x	x	x	x	x	x	
	Cytology		x		x		x		x		x		x	x	x	x	x	
ESMO	visit	x	x	x	x	x	x	x	x		x		x	x	x	x	x	
	CT Chest	x	x	x	x	x	x	x	x		(x)		x	(x)	x	(x)	x	
	CT Abd/Pelv	x	x	x	x	x	x	x	x		x		x	x	x	x	x	
	Cystoscopy	(x)	x	(x)	x	(x)	x	(x)	x	(x)	x	(x)	x	x	x	x	x	
	Cytology	?																
GERMAN S3 GUIDELINE	visit	x	x	x	x	x	x	x	x	x	x	x	x	x	x	x	x	Annually
	CT Chest	x	x		x		x		x		x		x		x		x	
	CT Abdomen	x	x		x		x		x		x		x		x		x	
	Cystoscopy	x	x	x	x	x	x	x	x	x	x	x	x	x	x	x	x	Annually
	Cytology	x	x	x	x	x	x	x	x	x	x	x	x	x	x	x	x	
NCCN	visit	x	x	x	x	x	x	x	x		x		x	x	x		x	Annually
	CT Chest (or chest x-ray)	(x)	X	(x)	x	(x)	x	(x)	x									
	CTU Abd/Pelv (or MRU)^a,b^	(x)	X	(x)	x	(x)	x	(x)	x				x		x		x	
	Cystoscopy	x	x	x	x	x	x	x	x		x		x	x	x		x	Annually
	Cytology		(x)		x		(x)		x									

^a^CTU/MRU image upper tracts + axial imaging of abdomen/pelvis only in years 1 + 2

^b^FDG PET/CT (category 2B) only if metastatic disease suspected

? investigation recommended without exact time schedule

*X’ and X’’* Bladder Cancer Canada recommends the same imaging studies and intervals for TMT as for cystectomy, there it differs between < / = pT2 (x), pT3-4 (X’) and pTxN + (X’’)

*(x)* Optional

### Ultrasound

Ultrasonography of the kidneys after trimodal treatment is not recommended by any guideline.

### Cystoscopy and cytology

Cystoscopy in patients who have undergone trimodal treatment for muscle-invasive bladder cancer can be challenging due to morphological alterations mainly caused by radiation effects. These alterations often make it difficult to differentiate between malignant changes in the bladder and post-radiation changes such as fibrosis, pseudopapillary inflammation, and epithelial denudation, which are commonly observed [21]. Compared to patients without prior radiotherapy to the bladder, the specificity and positive predictive value to find a relapse after radiotherapy were reported as 88 and 40%, while without radiotherapy they were 90 and 77%, respectively [22]. Similarly, the diagnostic accuracy of cytology is also modified in trimodal treatment patients with a limited sensitivity and specificity of 50 and 85% [22]. Nearly all patients show abnormal cytology early after treatment but it normalizes in over half of the patients within the first 3 months [23]. Remarkably, most studies do not differentiate between spontaneous and bladder wash cytology. A modelling study using prospectively collected data suggests that monitoring symptoms such as haematuria and urgency, alongside urine cytology, may effectively reduce the number of cystoscopies required during follow-up [24].

Although there is limited strong data to support routine cytology, guidelines recommend its use, albeit with variations in timing. The American Urological Association (AUA) suggests cystoscopy every 3 months during the first year, every 4–6 months in the second year, and every 6–12 months thereafter. Cytology is also recommended but without a specific time schedule [17]. The European Association of Urology (EAU) and the German S3-guidelines suggest cystoscopy with regular cytology every 3–4 months during the first 3 years and every 6 months until the 5th year [18]. Additionally, the German S3-guidelines mention that annual cystoscopies should be performed after the 5th year and that a biopsy from each suspicious cystoscopy finding should be taken [20]. The European Society for Medical Oncology (ESMO) recommends cystoscopies every 3–6 months for the first 5 years [19]. The National Comprehensive Cancer Network (NCCN) and Bladder Cancer Canada recommend cystoscopy every 3 months in years 1–2, every 6 months in years 3–4, and annually in years 5–10. Bladder Cancer Canada emphasizes urine cytology at every cystoscopy, while the NCCN recommends cytology only every 6–12 months in the first 2 years and thereafter only if clinically indicated [15, 25]. Biopsies of the tumor site are suggested in several institutional follow-up protocols [7, 8, 26–29], but not mentioned in any other guidelines.

### Emerging biomarkers

Emerging biomarkers offer significant promise in improving the precision of disease stage assessment, monitoring therapeutic responses, and reducing the frequency and costs of traditional follow-up procedures in bladder cancer management [30–34]. Promising biomarkers for recurrence detection represent tumour informed ctDNA (NCT05630131), Bladder CARE™ [35], UroAmp [36, 37], and microRNAs [38].

### Disease-specific risk factors for worse outcomes and recurrence

Numerous clinical variables like T-stage [7, 8], N-stage [39], cytology during follow-up [23] multifocality [40], completeness of TURBT [7, 26, 27], hydronephrosis [7, 41] or presence of different histological subtypes [41, 42] have an influence on the risk for recurrence.

### Disease-specific recurrence patterns

Within 2–10 years after trimodal treatment, 14–52% experience local recurrence in the bladder as the most common relapse site (11–36% non-muscle invasive, 3–16% muscle invasive), 15–35% distant metastases, and 13–16% nodal recurrence [7, 8, 10, 43–45]. Five and 10 years after trimodal treatment, non-muscle-invasive recurrence, muscle-invasive recurrence, and distant metastases were estimated to be 31 and 36, 13 and 14, and 31 and 35%, respectively [7]. Distant recurrence is found in the lungs in 6%, in the bones in 3–8%, the adrenal glands in 6%, in non-regional lymph nodes in 14%, and recurrence in regional pelvic lymph nodes in 3–8% of the cases [9, 10, 46]. The median time from completion of trimodal treatment to detection of local non-muscle invasive relapse is 2 years [47], with most muscle-invasive and metastatic recurrences occurring within the first 5 years of follow-up. Recurrences beyond 5 years are uncommon [7] but still possible: nearly 10% of all non-muscle invasive relapses occur later than 10 years after the initial treatment with complete response [28]. However, these late relapses may not only arise from recurrences but also from secondary cancers in the context of field cancerization.

### Functional follow-up/quality of life

Trimodal treatment has the potential to maintain overall quality of life, including aspects related to general well-being, body image, and the mitigation of negative effects, along with favorable outcomes concerning bowel and sexual quality of life [48, 49]. This is supported by bladder preservation rates of 60–87% at 5 years post-treatment, and 45–76% at 10 years [10, 27, 41]. Nonetheless, it is crucial to consider and discuss the side effects related to the treatment [3].

Common short-term genitourinary side effects, such as hematuria and dysuria, caused by radiation cystitis are generally of minor severity (CTCAE grades 1–2), affecting 20–65% of patients [9, 44]. Long-term toxicities of various severity grades, including diminished bladder capacity, urgency, incontinence, and urinary leakage, have been reported at rates of 25, 15, 19, and 19%, respectively, during follow-up [49]. Post-radiation urodynamic studies highlight significant compliance reduction and decreased maximum cytometric capacity [50, 51]. In addition, urinary tract infections are common after trimodal treatment [50, 51]. While severe complications like urethral strictures or hydronephrosis are uncommon [52], urinary symptoms such as urgency or incontinence are up to three times more prevalent in women compared to men [49]. Furthermore, late genitourinary toxicities of a significant severity (Grade 3–4) may differ among the different radiotherapy fractionation-regimens [43]. Only a minor proportion of patients report distress caused by gastrointestinal late complications with 9% of the affected patients reporting a moderate or greater distress caused by diarrhoea and 14% by bowel urgency [49]. In a study by Efstathiou et al. from 2009 looking at four RTOG trials, low rates of severe (≥ Grade 3) late pelvic (genitourinary or gastrointestinal) toxicities were seen for patients completing trimodal treatment and retaining their native bladder [29].

Based on long-term data from radiotherapy in prostate cancer reporting rates of fecal incontinence of up to 10%, there are concerns that similar or even worse outcomes could be observed after trimodal treatment, however there are only limited long-term data, especially from cohorts treated with modern radiotherapy techniques [53]. Therefore, novel radiotherapy techniques are explored including intensity-modulated radiotherapy (IMRT), the more recently developed volumetric-modulated arc therapy (VMAT) or even adaptive radiotherapy (ART); they have an improved dose homogeneity and sparing of the bladder, rectum, and pelvis and might reduce toxicities [54]. This potentially positive effect must be confirmed in comparative clinical trials. First results are promising as in a prospective trial only 20% of the patients suffered from acute grade ≥ 2 gastrointestinal toxicity and 16% had grade ≥ 2 genitourinary toxicities when receiving VMAT [55]. In addition, ART could further decrease toxicity [54]. Recently published results of the RAIDER Trial (NCT02447549) have shown that standard dose adaptive tumor focused radiotherapy (SART) and dose escalated adaptive tumor boost radiotherapy (DART) had lower Grade ≥ 2/3 late toxicity compared to standard whole bladder radiotherapy (WBRT). Grade ≥ 2/3 late toxicity rates 6–18 months after trimodal therapy were significantly reduced by DART (42%/9%, 35%/4%, and 32%/6% for WBRT, SART, and DART) [56].

## Discussion and conclusion

This systematic review examines trimodal treatment in the context of muscle-invasive bladder cancer, aggregating findings on follow-up assessments, the location and timing of recurrence stratified by disease stage and contrasting the follow-up protocols delineated in various guidelines. Our analysis reveals that existing follow-up recommendations are predicated on a limited evidence base. There is a clinical need to enhance follow-up personalization by tailoring it to an individual's recurrence risk. Additionally, there is scope for future research to integrate novel biomarkers, aiming to bolster oncological outcomes and curtail healthcare expenditures.

Trimodal treatment is commonly employed in two distinct populations: patients with adequate bladder function who prefer bladder preservation, and frail patients who are not suitable for surgery. Surprisingly, many studies demonstrate comparable outcomes of radical cystectomy versus trimodal treatment in patients who are medically fit enough for both treatment strategies, but in daily clinical practice trimodal treatment is often not even offered to all patients. but rather to the frail patient population only. In patients with adequate bladder function who prefer bladder preservation, there is an unmet need for research addressing the question of functional outcomes specifically dedicated to bladder related wellbeing and symptoms by means of patient reported outcome measures (PROMs/questionnaires), management of radiation-induced lower urinary tract dysfunction and the development of safe follow-up protocols enabling timely salvage cystectomy. For instance, investigating surveillance of the lower and upper urinary tract using innovative MRI protocols, CT, or ultrasonography or novel biomarkers should be explored. It should also be noted, that with the continuous innovation, and personalization of treatment strategies, such as the use of new radiotherapy techniques (IMRT, VMAT, adaptive radiotherapy), oncological outcomes as well as toxicity profiles could be improved. Consequently, the development of personalized surveillance protocols is needed to adapt to these evolving treatment approaches. In contrast, frail patients require a goal-directed surveillance approach that takes into account the limited therapeutic consequences and incorporates competing risks such as age, comorbidities (e.g., geriatric assessment, malnutrition) and other survival surrogates to offer individually tailored surveillance strategies based on their risk profiles.

In this systematic review focusing on follow-up strategies post-trimodal treatment for muscle-invasive bladder cancer, we highlighted the need for personalized follow-up approaches, given the current recommendations are based on limited evidence. Our examination suggests the potential for individualized protocols that factor in patient-specific risks of recurrence, coupled with the integration of novel biomarkers, to enhance oncological outcomes and optimize healthcare resources. Such tailored strategies promise not only to improve patient monitoring and intervention timeliness but also to contribute to cost-effective healthcare management in the context of bladder cancer post-treatment surveillance.

## Take home message

Guideline recommendations and institutional follow-up protocols after trimodal treatment only slightly differ regarding the modality, intensity, and timing of follow-up investigations, but are not personalized to the various risk factors for relapse, which can possibly lead to suboptimal care of bladder cancer survivors. More prospective trials are needed to assess how bladder cancer patients can be followed after trimodal treatment in order to ensure optimal oncological and functional outcomes. Furthermore, the role of PROMs/questionnaires and novel biomarkers during follow-up should be defined and new protocols implementing new therapeutic modalities such as adaptive radiotherapy and immunotherapies should be developed.

## Supplementary Information

Below is the link to the electronic supplementary material.

Supplementary file1 (PDF 304 KB)

Supplementary file2 (PDF 164 KB)

Supplementary file3 (PDF 250 KB)

## Data Availability

All data supporting the findings of this study are available within the paper and its Supplementary Information.
