# Supplementary material for: Follow-up strategies after trimodal treatment for muscle-invasive bladder cancer: a systematic review
Source: World J Urol. 2024 Sep 19;42(1):527. doi: 10.1007/s00345-024-05196-7 (PMC11413066; doi:10.1007/s00345-024-05196-7)
Supplement: Supplementary file 1 — Supplementary file1 (PDF 304 KB) [file 345_2024_5196_MOESM1_ESM.pdf]

Online Resource 3 (Supplementary Table 1: Included studies for review)

Follow-Up Strategies after Trimodal Treatment for Muscle-invasive Bladder Cancer: A systematic review - World Journal of Urology

Ernest Kaufmann, Stefanie Aeppli, Winfried Arnold, Panagiotis Balermpas, Jörg Beyer, Uwe Bieri, Richard Cathomas, Berardino De Bari, Marco Dressler, Daniel S. Engeler, Andreas Erdmann, Andrea Gallina, Silvia Gomez, Matthias Guckenberger, Thomas Hermanns, Lucca Ilaria, Hubert John, Thomas M. Kessler, Jan Klein, Mohamed Laouiti, David Lauffer, Agostino Mattei, Michael Müntener, Daniel Nguyen, Philipp Niederberger, Alexandros Papachristofilou, Lukas Prause, Karsten Reinhardt, Emanuela Salati, Philippe Sèbe, Mohamed Shelan, Räto Strebel, Arnoud J. Templeton, Ursula Vogl, Marian Severin Wettstein, Deborah Zihler, Thomas Zilli, Daniel Zwahlen, Beat Roth, Christian Fankhauser

Corresponding Author: Christian D. Fankhauser, Department of Urology, Luzerner Kantonsspital, Spitalstrasse 6000, 16 Lucerne, Switzerland (cdfankhauser@gmail.com) ORCID 0000-0002-4073-5488

title	Author	year of publication	country	sample size	observation period	stages	Chemotherapy	max. Radiation Dose	median follow up (months)	Follow-up Schedule	OS % (year)	PFS % (year)	CSS % (year)	Influence on OS (CSS)	Influence on PFS	Late Toxicity reported	Functional bladder survival % (year)	prospective/retrospective
Combined chemotherapy and radiation with selective organ preservation for muscle-invasive bladder carcinoma. A single- institution phase II study	G. Fellin	1997	Italy	56	1989-1994	T2-T4	two courses of methotrexate, cisplatin and vinblastine	65Gy	46	6-monthly intervals with clinical, radiological and laboratory studies. Patients who had retained their bladder underwent cystoscopy with biopsies under anaesthesia and urine cytology	55 (5)	x	59 (5)	x	x	yes	41 (5)	p
Bladder Preservation by Combined Modality Therapy for Invasive Bladder Cancer	L. A. Kachnic	1997	USA	106	1986-1993	T2-T4a	two cycles of chemotherapy (methotrexate, cisplatin, vinblastine [MCV]	65Gy	61	cystoscopy, biopsy of the tumor site, bimanual examination under anesthesia, and urine cytology every 3 months for 2 years and every 6 months thereafter. Follow-up abdominal and pelvic CT scans were performed after the initial 39.6 Gy of radiation, 3 months after the completion of therapy, and then every 6 to 12 months.	52 (5)	x	x	x	x	x	43 (5)	p
Combined-Modality Treatment and Selective Organ Preservation in Invasive Bladder Cancer: Long-Term Results	C. Rödel	2002	Germany	415	1982-2000	T1-T4	RT and consisted of cisplatin (25 mg/m /d; 30-minute infusion on 52 consecutive days) in 145 patients or carboplatin (65 mg/m /d; 30-minutes infusion on 5 consecutive days) in 95 patients with decreased creatinine clearance (□ 60 mL/min) or congestive heart disease	69Gy	36	3-month intervals for the first 2 years and every 6 months thereafter. Evaluations consisted of pertinent medical history, physical examination, complete blood counts and blood chemistry, urine cytology, and cystoscopy with biopsies of all suspected areas	51, 31 (5,10)	x	x	T-stage, incomplete TUR-B	x	yes	42,27 (5,10)	r
Conservative Treatment of Invasive Bladder Carcinoma by Transurethral Resection, Protracted Intravenous Infusion Chemotherapy, and Hyperfractionated Radiotherapy	D. T. Danesi	2004	Italy	77	1992-2001	T2-T4	MCV	69Gy	82	physical and instrumental examination every 3 months for the first 2 years, and every 6 months thereafter. Each year, thoracic and abdominopelvic CT scans, bone scans, and every other instrumental examination or laboratory test, if indicated,	59 (5)	x	75 (5)	x	x	yes	47 (5)	r

Conservative treatment with transm'ethral resection, neo-adjuvant chemotherapy followed by radiochemotherapy in stage T2-3 transitional bladder cancer	M. Cobo	2006	Spain	29	1996-2005	T2-T3	CMV (15 p) or Gemcitabine-Cisplatin (14 p)	65Gy	69	cystoscopy, biopsy of the tumor site and urine cytology every 5-4 months for 1.5-2 years, and them every 6 months. We also performed complete blood test every 6 months, abdomen ultrasound or abdomen tomography scan every 6 months, and a chest x-ray every 12 months.	72 (5,75)	x	x	x	x	x	60 (5)	r
Combined-modality therapy with gemcitabine and radiation therapy as a bladder preservation strategy: long-term results of a phase I trial	K. S. Oh	2008	USA	24	1998-2003	T2-T3	twice- weekly gemcitabine	60Gy	67	cystoscopy with bladder washings and cytology every 3 months for 2 years and CT scans at 3, 6, 9, 12, 18, and 24 months, with annual scans thereafter as part of standard of care.	76 (5)	x	82 (5)	x	x	x	62 (5)	p
Late pelvic toxicity after bladder-sparing therapy in patients with invasive bladder cancer: RTOG 89-03, 95-06, 97-06, 99-06	J. A. Efstathiou ,	2009	USA	157	1990-2002	T2-T4a	different protocolls	different protocolls	65	cystoscopy, biopsy of the tumor site, bimanual examination under anesthesia, and urine cytology every 3 months in the first year, every 3 to 4 months in the second year, every 6 months for 3 years, and then annually	x	x	x	x	x	yes	x	p
Bladder preservation multimodality therapy as BJUIan alternative to radical cystectomy for treatment of muscle invasive bladder cancer	A. M. Maarouf	2010	Egypt	33	2005-2006	T2-T3	three cycles of MVAC chemotherapy, 2–3 weeks after the tumour resectio	60Gy	12	cystoscopy and tumour site biopsy conducted every 3 months. Abdominopelvic computed tomography and chest X-ray were conducted every 6 months.	64 (1)	x	x	x	x	x	x	P
Combined modality treatment with bladder preservation for muscle invasive bladder cancer	M. A. Sabaa	2010	Egypt	104	1999-2008	T2-T3	Gemcitabine was given at a dose of 1,200 mg/m2 by intravenous infusion over 30 minutes at day 1 and day 8. Cisplatin was given at a dose of 70 mg/m2 over 6 hours at day 1 only. This cycle was repeated every 4 weeks for 3 cycles.	65Gy	71	In the first 2 years: Abdominopelvic CT and CXR were done every 6 months and cystoscopy was performed every 3 months. In the third and fourth years: Abdominopelvic CT, CXR and cystoscopy were obtained every 6 months. Thereafter: The previous investigations were repeated annually.	59 (5)	x	69 (5)	tumor stage, multifocality and grading	x	x	x	P
Outcome of Trimodality Protocol for invasive bladder cancer patients at Karachi, Pakistan	M. A. Tunio	2011	Pakistan	116	2006-2009	T2-T3	weekly cisplatinum 40mg/m2 weekly prior to radiotherapy for six dose	65Gy	36	cystoscopy 6 weeks after completion of CRT, and then every 3 months for first year and every 6 months for the following years	54 (3)	x	x	Complete response, T-stage, hydronephrosis	x	yes	52 (3)	P
Long-term results of two prospective bladder-sparing trimodality approaches for invasive bladder cancer: neoadjuvant chemotherapy and concurrent radio-chemotherapy	A. Zapatero	2012	Spain	80	1990-2010	T2-T4	3 cycles of MCV CT followed by re-evaluation under anesthesia. CT consisted of weekly cisplatin before RT exposure in 34 patients (20 mg/m2/d, 30-minute intravenous infusion, 2 days/week). Taxol was used in 5 patients with mild renal insuf- ficiency (50 mg/m2/d, 1 day per week, 6 hours before RT	64Gy	72	cystoscopy re-evaluation, biopsy of the tumor site and of all suspected areas, and urine cytology every 3 months for 2 years and every 6 months thereafter. Chest X-ray and abdominal and pelvis computed tomography or magnetic resonance imaging were performed 3 months after the completion of therapy, and then every 6 to 12 months. Exploration under anesthesia and TURBT was performed in patients with suspicion of relapse	73, 60 (5,10)	x	82,80 (5,0)	x	x	22% GU, 6% GI	66,46 (5,10)	P
Long-Term Outcomes of Selective Bladder Preservation by Combined-Modality Therapy for Invasive Bladder Cancer: The MGH Experience	J. A. Efstathiou	2012	USA	348	1986-2006	T2-T4a	different protocolls	different protocolls	92	cystoscopy, tumor-site rebiopsy, bimanual examination under anesthesia (EUA), urine cytology, and radiology [8]. Cystoscopic surveillance occurred every 3 mo in the first year, every 3 to 4 mo in the second year, every 6 mo for 3 yr, and then annually. After 1–2 yr, in	52,35,22 (5,10,15)	x	64,59,57 (5,10,15)	T-stage, incomplete TUR-B, hydronephrosis, complete response (OS, CSS)	x	x	60,45,36 (5,10,15)	p

										patients with negative evaluations, the biopsy and EUA were usually omitted if no worrisome office endoscopic findings were present								
ERCC1 and XRCC1 expression predicts survival in bladder cancer patients receiving combined trimodality therapy	S. Sakano	2013	Japan	186	1994-2009	T2-T4	cisplatin	63Gy	39	cystoscopic examination followed by washing cytology every 3 months for the first 5 years and every 6 months thereafter. Complementary examinations, including chest X-ray and/or CT scan, were performed every 6 months.	x	x	x	x	x	x	x	r
Hypofractionated intensity modulated radiation therapy in combined modality treatment for bladder preservation in elderly patients with invasive bladder cancer	G.A. Turgeon	2014	Canada	24	2008-2012	T2-T3	concomitant weekly gemcitabine 100 mg/m2 or cisplatin 40 mg/m2	50Gy	28	cystoscopy was performed every 3 months for the first 2 years and every 6 months thereafter. CT scan imaging was performed at 6 and 18 months after treatment and afterward as clinically indicated	69,61 (2,3)	x	80, 71 (2,3)	x	x	yes	x	r
Trimodality bladder-sparing approach without neoadjuvant chemotherapy for node-negative localized muscle-invasive urinary bladder cancer resulted in comparable cystectomy-free survival	C. Y. Lee	2014	China	70	2004-2012	T2-T4	cisplatin (30 mg/m2/week) for patients without impaired renal function defined as glomerular filtration rate (GFR) >50mL/min, or carboplatin (100 mg/m2 on days 1, 15, 31) for patients with renal function impairment.	67Gy	24	3-month intervals for the first 3 years and every 6 months thereafter. Post-treatment follow-up consisted of pertinent medical history, physical examination, urine cytology, cystoscopy, and radiological evaluation as clinically indicated	66 (2)	52 (2)	x	age, performance status, Complete response	x	yes	64 (2)	r
Long-term outcomes in patients with muscle-invasive bladder cancer after selective bladder-preserving combined-modality therapy: a pooled analysis of Radiation Therapy Oncology Group protocols 8802, 8903, 9506, 9706, 9906, and 0233	R. H. Mak	2014	USA	468	1988-2007	T2-T4a	6 different protocols	6 different protocols	52	cystoscopy, tumor site biopsy, bimanual examination under anesthesia, and urine cytology every 3 months for the first year, and then cystoscopy and cytology every 3 to 4 months during the second year, every 6 months for 3 years, and then annually.	57, 36 (5, 10)	x	71, 65 (5, 10)	hydronephrosis, T-stage, incomplete TUR, age (OS) hydronephrosis, T-stage (CSS)	x	x	x	P
Outcomes of trimodality approach in the management of T2N0M0 bladder cancer	B. Uysal	2015	Turkey	38	2008-2013	T2a/b	Cisplatin 70 mg/m2 was administered concomitantly in the 1st, 22nd and 43rd days of RT	60-66Gy	25	Cystoscopy was done by an urological surgeon. If a complete response was accomplished, the patients were followed up every 3 months for 3 years, every 6 months for the third to fifth years and annually thereafter. If a partial response or recurrence occurred, the cystectomy option was used. Follow-up examination included cystoscopy and biopsies of pretherapy tumor site, CT of whole abdominal region, chest X-ray, whole-blood test and urine cytology	64 (3)	x	73(3)	x	x	yes	x	r
Long-Term Outcomes Among Patients Who Achieve Complete or Near-Complete Responses After the Induction Phase of Bladder-Preserving Combined-Modality Therapy for Muscle-Invasive Bladder Cancer: A Pooled Analysis of NRG Oncology/RTOG 9906 and 0233	T. Mitin	2016	USA	119	1999-2008	T2-T4	different protocols	different protocols	71	cystoscopy, biopsy of the tumor site, bimanual ex- amination under anesthesia, and urine cytology every 3 months in the first year, every 3 to 4 months in the second year, every 6 months for 3 years, and then annually	72 (5)	68(5) only bladder recurrence	x	x	x	x	x	p
Quality of Life in Long-term Survivors of Muscle-Invasive Bladder Cancer	K. S. Mak	2016	USA	226	1990-2011	T2-T4	x	64Gy	67	6 validated instruments/questionnaires	x	x	x	x	x	x	x	r

Propensity Score Analysis of Radical Cystectomy Versus Bladder-Sparing Trimodal Therapy in the Setting of a Multidisciplinary Bladder Cancer Clinic	G. S. Kul-karni	2017	Canada	56	2008-2012	>T2-T3	cisplatin chemo- therapy (40 mg/m2) was administered weekly	64Gy	54	quarterly visits in year 1, biannual visits in years 2 and 3, and annual visits thereafter). Visits involved history, physical examination, blood work, and urinary cytology. CT or MRI bi- annually to detect recurrent disease and complica-tions. Patients who received TMT also underwent cystoscopy	x	x	77 (5)	x	x	x	x	r
Long-term single-institute ex-perience with trimodal blad-der-preserving therapy with proton beam therapy for mus-cle-invasive bladder cancer	E. Ta-kaoka	2017	Japan	70	1990-2015	T2-T3	methotrexate 30 mg/m2 and cisplatin 50 mg/m2 were ad-ministered half-and-half con-tinuously over a 2-hour period each in three courses in weeks 1, 4 and 7 through the cathe-ters placed in the bilateral in-ternal iliac arteries	41Gy	41	cystoscopy, urine cytology and chest X-ray every 3 months for the first year after the therapy, and every 6 months for 5 years thereaf-ter. Chest to pelvic CT was also performed every 6 months. We evaluated acute and late toxicities associated with treatment accord-ing to according to the US Na-tional Cancer Institute's Common Terminology Criteria for Adverse Events, version 4	90,82, 78 (3,5,10)	80,77,73 (3,5,10)	x	x	x	yes	x	r
Clinical Outcomes of Patients with Histologic Variants of Urothelial Cancer Treated with Trimodality Bladder-sparing Therapy	R.E. Kras-now	2017	USA	303	1993-2013	T2-T4a	different protocols	differ-ent pro-tocols	72	Patients were followed with cys-toscopy, cytology, and cross-sec-tional imaging. Surveillance oc-curred every 3 mo in the 1st yr, 3–4 mo in the 2nd yr, every 6 mo in the 3rd yr, and annually thereafter	61,42 (5,10)	x	75, 67 (5,10)	Age, T-stage, response to CR, hydronephrosis, CIS, incomplete TUR (OS), hy-dronephrosis, response to CR (CSS)	x	x	81, 76 (5,10)	r
Long-term Outcomes After Bladder-preserving Tri-mo-dality Therapy for Patients with Muscle-invasive Blad-der Cancer: An Updated Analysis of the Massachu-setts General Hospital Expe-rience	N. J. Gia-calone	2017	USA	475	1986-2013	T2-T4a	different protocols	65Gy	55	serial cystoscopy, tumor site re-biopsy, bimanual examination un-der anesthesia (EUA), urine cytol-ogy, and radiology, as delineated by their respective protocol. Cys-toscopy was performed approxi-mately every 3 mo for 1 yr, every 3–4 mo during Year 2, every 6 mo for Years 3 through 5, and annu-ally thereafter. An EUA and re-biopsy were performed during the 1st 6 mo of follow-up	57,39, 25 (5, 10, 15)	x	66,59,56 (5,10,15)	age, T-stage, re-sponse to CR, hydronephrosis, CIS, incomplete TUR (OS), T-stage, response to CR, CIS (CSS)	x	x	71, 69 (5, 10)	P
Selective bladder preserva-tion by combined modality protocol treatment: long-term outcomes of 190 patients with invasive bladder cancer	W. U. Shipley	2018	USA	190	1986-1997	T2-T4a	different protocols	65Gy	80	cystoscopy, biopsy of the tumor site, bimanual examination under anesthesia, and urinary cyto- logic examination every 3 months for 2 years. After 2 years, in patients with negative evaluations, the cold-cup biopsy was usually omit-ted if no worrisome endoscopic findings. The upper tracts were evaluated by serial computed to-mography and ureteral cytologic analysis and endoscopy when indi-cated. were present. Surveillance continued every 6 months for 3 more years and yearly thereafter.	54, 36 (5,10)	x	63,59 (5,10)	x	x	x	x	p
Incidence, Clinicopathologi-cal Risk Factors, Manage-ment and Outcomes of	A. Sanchez	2018	USA	342	1986-2013	T2-T4a	changing protocols during study	64-65Gy	64	examination under anesthesia, cys-toscopy with biopsy of the initial tumor site and visibly concerning	54	x	x	x	x	yes	x	r

Nonmuscle Invasive Recurrence after Complete Response to Trimodality Therapy for Muscle Invasive Bladder Cancer										lesions, and urine cytology after induction and consolidation chemoradiation. Thereafter office cystoscopic surveillance and urine cytology were per- formed every 3 months for the first 2 years, every 6 months for years 3 to 5 and yearly for life thereafter. Surveillance also included axial imaging of the chest, abdomen and pelvis.								
Long-term Outcomes and Patterns of Failure Following Trimodality Treatment With Bladder Preservation for Invasive Bladder Cancer	D. Büchser	2019	Spain	90	1990-2016	T2-T4	1) 3 cycles of neoadjuvant methotrexate-cisplatin-vinblastine (MCV), 2) concurrent weekly cisplatin (40 mg/m2/iv), 3) 6 cycles of concomi- tant weekly cisplatin	60-66Gy	94	cystoscopy, biopsies of any suspected areas, and urine cytology every 3 months for 2 years after treatment, every 6 months for 5 years, and then annually for a mini- mum of 10 years. Chest X-ray and abdominal and pelvis computed tomography scans were performed 3 months after the completion of therapy, every 6 months for 5 years, and annually thereafter.	67, 43 (5, 10)	x	81, 76 (5, 10)	≥ T2 bladder relapse	x	Urinary (20%), GI (7%) (>Grade 2)	x	r
Comparison of Costs of Radical Cystectomy vs Trimodal Therapy for Patients With Localized Muscle-Invasive Bladder Cancer	S. B. Williams	2019	USA	728	2002-2018	T2-T4a	x	x	x	x	x	x	x	x	x	x	x	r
The updated outcomes of bladder-preserving trimodal therapy using a real-time tumor-tracking radiotherapy system for patients with muscle-invasive bladder cancer	H. Miyata	2020	Japan	38	1998-2016	T2-T4	nedaplatin chemotherapy (70 mg/m2 intravenously) on days 1, 22 and 50	65Gy	28	physical examination, laboratory evaluations, urinary cytological tests and cystoscopy every 3 months for 2 years, then every 6 months for 3 years, and annually or more frequently thereafter	63,55,41 (3,5,10)	43,43,23 (3,5,10)	70,62,51 (3,5,10)	performance status, sex	histology	yes	100,100,75 (3,5,10)	r
Radiation with concurrent radiosensitizing capecitabine tablets and single-dose mitomycin-C for muscle-invasive bladder cancer: A convenient alternative to 5-fluorouracil	C. S. Voskuilen	2020	Netherlands	71	2014-2019	T2-T4a	Mitomycin-C was administered intravenously on day one at a dose of 12 mg/m2 with a maximum dose of 20 mg. Capecitabine was given twice daily at a dose of 825 mg/m2 throughout radiotherapy, excluding weekends	60Gy	23	cystoscopic evaluation every 3 months and abdominal/pelvic CT at 6 months follow-up, followed by every six months	85(2)	x	x	x	x	see table in publication	x	r
Trimodal therapy in T2-4aN0M0 bladder cancer—How to select the best candidate?	O. N. Gofrit	2020	Israel	105	2000-2019	T2-T4a	Cisplatin, at a dose of 40 mg/m2 weekly	62Gy	29	evaluation of kidney function, cystoscopy and CT of the chest, abdomen and pelvis at 3 and 6 months post-therapy completion, followed by 6 month in- tervals for 3 years, and then, at the clinician's discretion	60, 33 (2,5)	x	68, 45 (2,5)	age, tumor diameter	tumor diameter	x	x	r
Patient-reported Quality of Life Outcomes in Patients Treated for Muscle-invasive Bladder Cancer with Radiotherapy ± Chemotherapy in the BC2001 Phase III Randomised Controlled Trial	R. A. Huddart	2020	UK	458	2001-2008	T2-T4a	o chemotherapy received intravenous mitomycin C (12mg/m2) on day 1 of radiotherapy and continuous infusion of 5-fluorouracil (5-FU) 500 mg/m2/ 24 h for 5 days during radiotherapy fractions 1–5 and 16–20	55-64Gy	x	Functional Assessment of Cancer Therapy—Bladder (FACT-BL) questionnaires at baseline, end of treatment (EoT), and 6, 12, 24, 36, 48, and 60 months after radiotherapy	x	x	x	x	x	x	x	r

Gemcitabine based trimodality treatment in patients with muscle invasive bladder cancer: May neutrophil lymphocyte and platelet lymphocyte ratios predict outcomes?	P. Hurmuz	2021	Turkey	44	2005-2018	T2-T4a	weekly concurrent gemcitabine of 50 mg/m2	60Gy	21	cystoscopy was performed at 6, 9, and 12 months after the treatment and annually for up to 5 years. Physical examination, urine cytology, hematologic and biochemical analysis, chest, abdomen, and pelvis CT was performed every 3 months in the first 2 years, every 6 months for the next 3 years and annually thereafter	86, 64 (1, 2)	65, 44 (1,2) local rec 68, 48 (1,2) distant met.	88, 66 (1,2)	age, hydronephrosis (OS), age hydronephrosis, CIS (CSS)	x	urinary (20%) grade 2 acute, urinary (5%) grade 2 late	x	r
Neoadjuvant Chemotherapy-Guided Bladder-Sparing Treatment for Muscle-Invasive Bladder Cancer: Results of a Pilot Phase II Study	H. Shi	2021	China	59	2015-2018	T2-T4a	neoadjuvant gemcitabine/cisplatin + in responders concurrent cisplatin (40 mg/m2, once per week)	65Gy	45	Physical examination, blood sampling, and imaging every 3 months for 2 years after combined modality treatments, every 6 months in the third year, and annually afterward. A questionnaire assessment with the 36-item Short Form Health Survey (SF-36) was used for all of the patients. The Overactive Bladder Symptom Score (OABSS) was used for patients who underwent bladder-sparing treatment at the 6th month after enrollment	88 (3)	74 (3)	x	x	x	x	x	P
The Effect of Tri-Modality Therapy with Bladder Preservation for Selective Muscle-Invasive Bladder Cancer	Z. Zhiyu	2021	China	79	2012-2015	T2-T4a	Intravenous chemotherapy including gemcitabine 1000 mg/m2 (day 1 and day 8), cisplatin 70 mg/m2 (day 2) was performed within 2 to 4 weeks after the surgery, with a cycle of 3 weeks, a total of 3 cycles	24-25Gy	48	very 3 months within 2 years, every 6 months within 2 to 5 years, and once a year after 5 years. The reexamination included a chest x-ray and abdominal and pelvis CT or MRI. For patients who received TMT, cystoscopy, and urine cytology should be taken every 3 months for 2 years, every 6 months for 5 years, and annually for at least 10 years.	95, 80, 33 (1,2,5)	75, 65, 34 (1,2,5)	x	Age, TNM, PNI	PNI (prognostic nutritional index)	x	x	r
Characterization and management of NMIBC recurrences after TMT: a matched cohort analysis	K. Ajib	2021	Canada	124	2008-2019	T2-T4	MVAC (Methotrexate, Vinblastine, Doxorubicin, and Cisplatin), CMV (Cisplatin, Methotrexate, and Vinblastine), GC (Gemcitabine and Cisplatin), or Gemcitabine alone with localizing lipidol injections as a fiducial marker. In our series, GC was the most common regimen used	64-66	43	TMT patients are followed-up every 3 months for 2 years with a chest abdomen pelvis CT scan and a cystoscopy, then every 6 months for 2 years, then annually.	70 (2)	91, 75 (1,2)	x	x	x	x	100, 85 (1,2)	r
Long-term outcomes after bladder-preserving tri-modality therapy for patients with muscle-invasive bladder cancer	E. Fabiano	2021	France	313	1988-2013	T2-T3	5-FU/cis- platin concomitantly with radiation. Cisplatin (15 mg/m2/d) and 5-FU (400mg/m2/d) were administered as a short infusion over 2h o days 1,2,3,15,16, and 17. On days 1,3, 15, and 17, chemotherapy infusion was begun 2h before irradiation	45Gy	59	Clinical examination, urine cytology, cystoscopy (with biopsy if necessary), thoracoabdominopelvic CT scan were per- formed routinely every 6 months or at the recurrence of symptoms.	69,62 (5,7)	x	x	age, Complete response	x	yes	69,65 (5,7)	r
The efficacy of trimodal chemoradiotherapy with gemcitabine and cisplatin as a bladder-preserving strategy for the treatment of muscle-invasive bladder cancer: a single-arm phase II study	K. Kobayashi	2022	Japan	35	2011-2018	T2-T4	gemcitabine 300 mg/m2 infused for 30 min and cisplatin 30 mg/m2 infused for 2 h on the same day. They were administered once per week on days 1, 8, 15, 22, 29 and 36	54Gy	51	every 3 months for cystoscopy and every 2 years for urine cytology, following every 6 months for up to 5 years, and every 6 months for computed tomography scan for 5 years. After 5 years, examinations were performed at the discretion of each attending physician	88, 75 (3, 5)	x	90, 85 (3, 5)	x	x	yes	x	P

Morphologic alterations post trimodal therapy in muscle-invasive urothelial carcinoma: understanding the impact of post-treatment changes on the pathological interpretation and their potential clinical correlates	G. Evaristo	2022	Canada	73	2004-2018	T2-T3	x	x	28	combination of imaging, cystoscopy, and urine cytology. Biopsies through TUR when deemed indicated by a treating urologist based on cytologic/ cystoscopic findings and clinical context. Follow-up was performed every 3-4 months in the first 2 years, every 6 months in the subsequent 3 years, followed by annual surveillance	x	x	x	Fibrosis (CSS)	x	x	x	r
Bladder preservation in older adults with muscle-invasive bladder cancer: A retrospective study with concurrent chemotherapy and twice-daily hypofractionated radiotherapy schedule	A. Alati	2022	France	85	1988-2017	T2-T3	5-FU (400 mg/m /day) and cisplatin (15 mg/m2/day) and was given on days 1, 2, and 3 of each series 2 h before radiotherapy sessions	44Gy	63	Follow-up consisted of clinical examination, cystoscopy, abdominopelvic scanner, and/or ultrasound every 6 months for 5 years, then once a year. Urinary cytology was not systematic	94, 61 (1,5)	x	98, 78 (1,5)	Malnutrition	x	yes	median (113) 89, 70 (1,5)	r
The prognostic value of urinary cytology after trimodal therapy (TMT) for muscle-invasive bladder cancer	L. C. McLoughlin	2022	Canada	129	2002-2017	T2-T4	X	X	40	surveillance included cystoscopy, cytology, and cross-sectional imaging. Cytology and cystoscopy were performed at 3-monthly intervals for years 1 and 2, 6-monthly for years 3 to 5, extending to yearly intervals thereafter.	x	x	x	x	x	x	x	r
Impact of sarcopenia on outcomes of patients treated with trimodal therapy for muscle invasive bladder cancer	A. Almarzouq	2022	Canada	141	2002-2018	T2-T4	Gemcitabine alone (weekly at a dose of 100 mg/m2), was the main systemic agent used (66.7%) followed by cisplatin alone (weekly at a dose of 40 mg/m2) as the second most common drug (19%)	50Gy	32	Follow-up was performed with 3 to 6 months interval with urine cytology, cystoscopy, and cross-sectional imaging.	52.5 (5)	x	x	x	x	x	x	r
Long term cost comparisons of radical cystectomy versus trimodal therapy for muscle-invasive bladder cancer	V. Golla	2022	USA	651	2002-2009	T2-T4a	x	x	x	x	x	x	x	x	x	x	x	r
Utilising alternative cystoscopic schedules to minimise cost and patient burden after trimodality therapy for muscle-invasive bladder cancer	R. Krishnarty	2023	India	112	2009-2019	T1-T4	cisplatin 30 mg/m2 or gemcitabine 75–100mg/m2 given once weekly	64Gy	19	three- monthly for the first 2years, six-monthly for the next 3years, and yearly after that. A TA consisting of clini- cal examination, urine cytology and cystoscopy was per- formed at every follow-up	x	x	x	x	x	x	x	r
