# Supplementary material for: Follow-up strategies after trimodal treatment for muscle-invasive bladder cancer: a systematic review
Source: World J Urol. 2024 Sep 19;42(1):527. doi: 10.1007/s00345-024-05196-7 (PMC11413066; doi:10.1007/s00345-024-05196-7)
Supplement: Supplementary file 2 — Supplementary file2 (PDF 164 KB) [file 345_2024_5196_MOESM2_ESM.pdf]

Online Resource 1(Supplementary File 1: Search algorithm)

Follow-Up Strategies after Trimodal Treatment for Muscle-invasive Bladder Cancer: A systematic review - World Journal of Urology

Ernest Kaufmann, Stefanie Aeppli, Winfried Arnold, Panagiotis Balermpas, Jörg Beyer, Uwe Bieri, Richard Cathomas, Berardino De Bari, Marco Dressler, Daniel S. Engeler, Andreas Erdmann, Andrea Gallina, Silvia Gomez, Matthias Guckenberger, Thomas Hermanns, Lucca Ilaria, Hubert John, Thomas M. Kessler, Jan Klein, Mohamed Laouiti, David Lauffer, Agostino Mattei, Michael Müntener, Daniel Nguyen, Philipp Niederberger, Alexandros Papachristofilou, Lukas Prause, Karsten Reinhardt, Emanuela Salati, Philippe Sèbe, Mohamed Shelan, Räto Strebel, Arnoud J. Templeton, Ursula Vogl, Marian Severin Wettstein, Deborah Zihler, Thomas Zilli, Daniel Zwahlen, Beat Roth, Christian Fankhauser

Corresponding Author: Christian D. Fankhauser, Department of Urology, Luzerner Kantonsspital, Spitalstrasse 6000, 16 Lucerne, Switzerland (cdfankhauser@gmail.com) ORCID 0000-0002-4073-5488

PICO

**Population**	Patients with bladder cancer
**Intervention**	Trimodal therapy
**Comparison**	Protocols of different follow-ups
**Outcome**	Any oncological or functional outcome

Definitive Search 22.02.2024 on Pubmed

Additional filters: English, from 1990 – until 22nd of February 2024

(
("Urinary Bladder Neoplasms"[Mesh])
OR
(
(urin*[Title/Abstract])
AND
(bladder[Title/Abstract])
AND
(Cancer*[Title/Abstract] OR carcinoma*[Title/Abstract] OR neoplasm*[Title/Abstract] OR maligna*[Title/Abstract] OR tumor*[Title/Abstract] OR tumour*[Title/Abstract])
)
AND
(trimodal[Title/Abstract] OR trimodality[Title/Abstract] OR “tri modality”[Title/Abstract] OR “tri-modality”[Title/Abstract] OR TMT[Title/Abstract] OR “combined-modality therapy” [Title/Abstract]
)
AND
(Follow*[Title/Abstract] OR “Follow up”[Title/Abstract] OR Follow up*[Title/Abstract] OR surveillance[Title/Abstract] OR outcome[Title/Abstract] OR response[Title/Abstract] OR survivor*[Title/Abstract])
)
NOT
("case report"[Title/Abstract] OR review[Title] OR “rats”[Title/Abstract] OR “dogs”[Title/Abstract] OR “cats”[Title/Abstract] OR “animal”[Title/Abstract])

**Bladder cancer**	**Trimodal therapy**	**Follow-Up**	**Results**
X			52.958
	X		9.277
		X	5.981.245
X	X	X	196
