# Supplementary material for: Follow-up strategies after trimodal treatment for muscle-invasive bladder cancer: a systematic review
Source: World J Urol. 2024 Sep 19;42(1):527. doi: 10.1007/s00345-024-05196-7 (PMC11413066; doi:10.1007/s00345-024-05196-7)

Online Resource 2 (Supplementary Figure 1: Study Selection Process Flow Chart)

Follow-Up Strategies after Trimodal Treatment for Muscle-invasive Bladder Cancer: A systematic review - World Journal of Urology

Ernest Kaufmann, Stefanie Aeppli, Winfried Arnold, Panagiotis Balermpas, Jörg Beyer, Uwe Bieri, Richard Cathomas, Berardino De Bari, Marco Dressler, Daniel S. Engeler, Andreas Erdmann, Andrea Gallina, Silvia Gomez, Matthias Guckenberger, Thomas Hermanns, Lucca Ilaria, Hubert John, Thomas M. Kessler, Jan Klein, Mohamed Laouiti, David Lauffer, Agostino Mattei, Michael Müntener, Daniel Nguyen, Philipp Niederberger, Alexandros Papachristofilou, Lukas Prause, Karsten Reinhardt, Emanuela Salati, Philippe Sèbe, Mohamed Shelan, Räto Strebel, Arnoud J. Templeton, Ursula Vogl, Marian Severin Wettstein, Deborah Zihler, Thomas Zilli, Daniel Zwahlen, Beat Roth, Christian Fankhauser

Corresponding Author: Christian D. Fankhauser, Department of Urology, Luzerner Kantonsspital, Spitalstrasse 6000, 16 Lucerne, Switzerland (cdfankhauser@gmail.com) ORCID 0000-0002-4073-5488

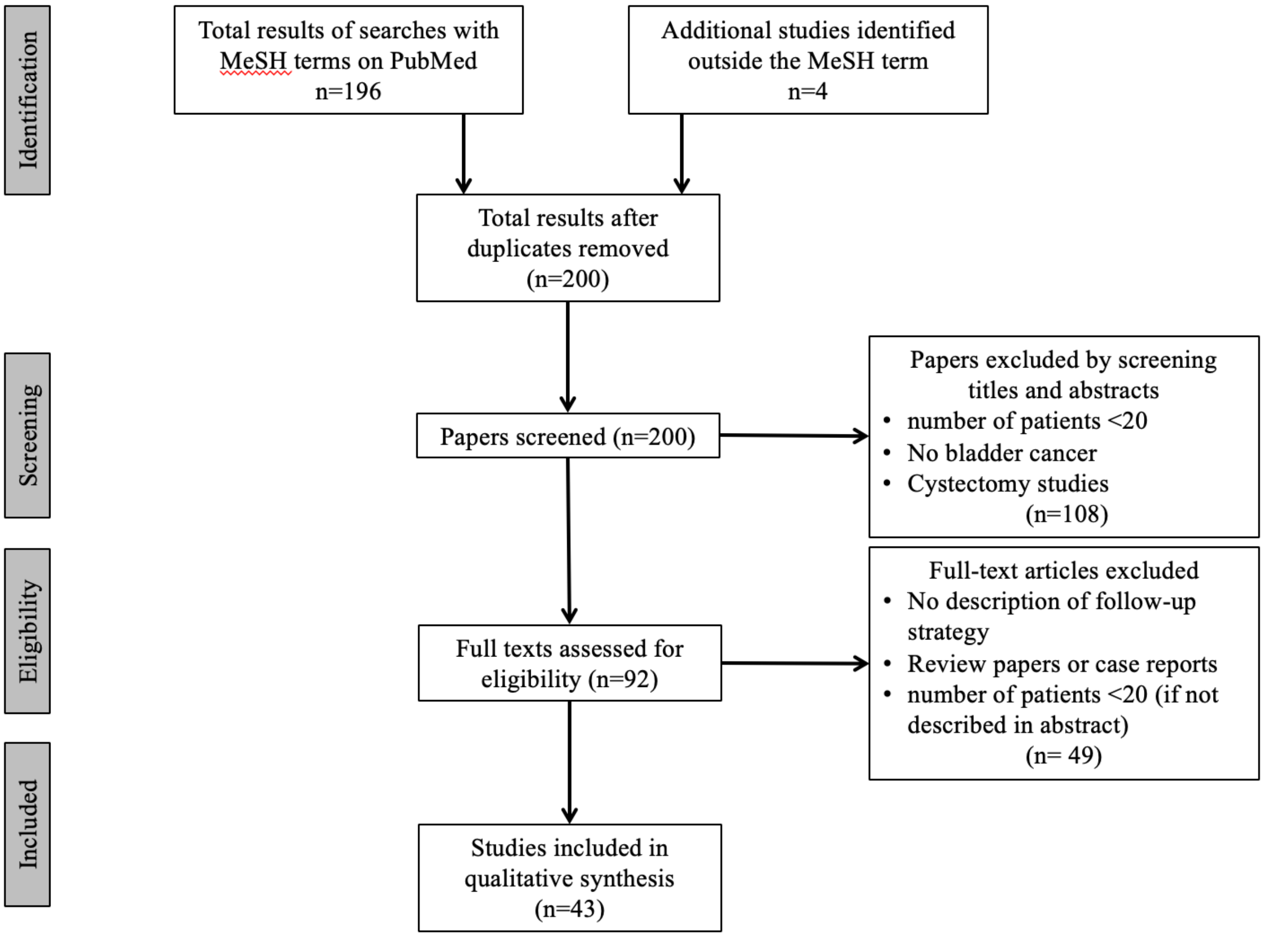

Supplement: Supplementary file 3 — Supplementary file3 (PDF 250 KB) [file 345_2024_5196_MOESM3_ESM.pdf]
